# Validation of a questionnaire for central nervous system aspects of joint pain: the CAP questionnaire

**DOI:** 10.1093/rheumatology/keae342

**Published:** 2024-06-18

**Authors:** Daniel F McWilliams, Vasileios Georgopoulos, Jayamala Patel, Bonnie Millar, Stephanie L Smith, David A Walsh

**Affiliations:** Pain Centre Versus Arthritis, University of Nottingham, Nottingham, UK; NIHR Nottingham Biomedical Research Centre, University of Nottingham, Nottingham, UK; Academic Unit of Injury, Recovery and Inflammation Sciences, University of Nottingham, Nottingham, UK; Pain Centre Versus Arthritis, University of Nottingham, Nottingham, UK; Academic Unit of Injury, Recovery and Inflammation Sciences, University of Nottingham, Nottingham, UK; Pain Centre Versus Arthritis, University of Nottingham, Nottingham, UK; Academic Unit of Injury, Recovery and Inflammation Sciences, University of Nottingham, Nottingham, UK; Pain Centre Versus Arthritis, University of Nottingham, Nottingham, UK; NIHR Nottingham Biomedical Research Centre, University of Nottingham, Nottingham, UK; Academic Unit of Injury, Recovery and Inflammation Sciences, University of Nottingham, Nottingham, UK; Pain Centre Versus Arthritis, University of Nottingham, Nottingham, UK; Academic Unit of Injury, Recovery and Inflammation Sciences, University of Nottingham, Nottingham, UK; Pain Centre Versus Arthritis, University of Nottingham, Nottingham, UK; NIHR Nottingham Biomedical Research Centre, University of Nottingham, Nottingham, UK; Academic Unit of Injury, Recovery and Inflammation Sciences, University of Nottingham, Nottingham, UK; Department of Rheumatology, Sherwood Forest Hospitals NHS Foundation Trust, Sutton-in-Ashfield, UK

**Keywords:** pain, epidemiology, osteoarthritis, lower back pain, fibromyalgia, nociplastic pain, central sensitization

## Abstract

**Background:**

Neuropathic-like pain, fatigue, cognitive difficulty, catastrophizing, anxiety, sleep disturbance, depression and widespread pain associate with a single factor in people with knee pain. We report the Central Aspects of Pain questionnaire (CAP) to characterize this across painful musculoskeletal conditions.

**Methods:**

CAP was derived from the 8-item CAP-Knee questionnaire, and completed by participants with joint pain in the Investigating Musculoskeletal Health and Wellbeing survey. Subgroups had OA, back pain or FM. Acceptability was evaluated by feedback and data missingness. Correlation coefficients informed widespread pain scoring threshold in relation to the other items, and evaluated associations with pain. Factor analysis assessed CAP structure. Intraclass Correlation Coefficient (ICC) between paper and electronic administration assessed reliability. Friedman test assessed score stability over 4 years in people reporting knee OA.

**Results:**

Data were from 3579 participants (58% female, median age 71 years), including subgroups with OA (*n* = 1158), back pain (*n* = 1292) or FM (*n* = 177). Across the three subgroups, ≥10/26 painful sites on the manikin scored widespread pain. Reliability was high [ICC = 0.89 (95% CI 0.84–0.92)] and CAP scores fit to one- and two-factor model, with a total CAP score that was associated with pain severity and quality (r = 0.50–0.72). In people with knee pain, CAP scores were stable over 4 years at the group level, but displayed significant temporal heterogeneity within individual participants.

**Conclusions:**

Central aspects of pain are reliably measured by the CAP questionnaire across a range of painful musculoskeletal conditions, and is a changeable state.

Rheumatology key messages

- The Central Aspects of Pain (CAP) questionnaire reliably measures a construct associated with pain across a range of musculoskeletal conditions.
- The CAP questionnaire is a candidate measurement tool for nociplastic pain in musculoskeletal conditions.

## Introduction

Chronic pain is a symptom shared across many musculoskeletal conditions, even when disease management has been optimized. Musculoskeletal pathology is an important treatment target, but often does not adequately explain pain or its persistence. Processing of nociceptive signals in the spinal cord can increase pain severity, exacerbated by inadequate descending inhibitory control from the brainstem. Central sensitization is an increased responsiveness of CNS neurons to a standardized nociceptive input. Changes in brain connectivity might explain increased emotional components of pain. Pain, increased by these neuronal mechanisms, both in severity and distribution, beyond that explained by musculoskeletal pathology, has been called ‘nociplastic’ [1]. Measurement of these CNS aspects of pain is a prerequisite for understanding their mechanistic underpinning, and their ability to predict future pain and responses to treatment.

Chronic pain is associated with CNS dysfunction across several domains, depression, anxiety, catastrophizing, impaired cognitive function, sleep disturbance and fatigue [2]. Chronic musculoskeletal pain may take on neuropathic-like characteristics, and may spread beyond index sites [3]. Scores from questionnaires that capture these symptoms correlate with each other, and with quantitative sensory testing (QST) indices of central sensitization [4]. Although it is challenging to measure mechanisms via questionnaire, these shared associations might indicate diverse consequences of pain or a more general CNS dysfunction. We previously reported a questionnaire measuring central aspects of knee pain (Central Aspects of Pain Knee, CAP-Knee) which was designed with the aim to predict outcomes and stratify patients [5]. CAP-Knee comprises eight self-reported items associated with depression, anxiety, catastrophizing, cognition, sleep, fatigue and a body pain manikin.

No ‘gold standard’ exists for measuring central sensitization or nociplastic pain, providing challenges for questionnaire validation. Both QST and questionnaires depend on self-report, acknowledging that pain is a subjective experience. Objectivity is maximized by validated and standardized questionnaires and nociceptive stimuli in QST. Various QST modalities reflect different pain mechanisms, such as spinal sensitization (Temporal Summation) or descending inhibitory or facilitatory control of nociceptive transmission (Conditioned Pain Modulation). Different QST modalities sometimes only weakly correlate with each other [6], underlining the heterogeneous CNS mechanisms that modulate pain. In the absence of a ‘gold standard’, measurement tools are validated against multiple (and sometimes differing) criteria, which, in turn, inform interpretation of measured outcomes.

The CAP-Knee questionnaire measures a unitary overarching factor that was associated with sensitivity, as measured by pressure pain detection thresholds (PPT) distal to the index knee [7]. It predicted persistent pain in a cohort of people with knee pain more strongly than did any individual characteristic measured by questionnaires from which CAP-Knee items were derived [8]. We here refer to that underlying factor as Central Aspects of Pain factor (CAPf). Identification of CAPf is consistent with (although not proof of) a condition of CNS dysfunction in people with nociplastic pain.

That self-reported symptoms can be used to measure aspects of CNS pain processing is supported by data using other questionnaires. Widespread pain distribution is associated with pain severity and QST evidence of central pain sensitivity [9]. Pain distribution is addressed by questionnaires such as the 9-item Central Sensitization Inventory (CSI9) [10, 11] and 8-item Somatic Symptom Scale (SSS8) [12], each of which addresses frequently co-existing medically unexplained symptoms, and is also associated with QST evidence of central pain sensitivity [13–15]. Pain distribution items addressing widespread or specific body site pain comprise five (55%) of the items in CSI9, and five (63%) items in SSS8. Unlike CSI9 and SSS8, CAP-Knee was derived in a musculoskeletal pain population, and addresses a broader range of cognitive and affective factors, each of which has been associated with QST evidence of central pain sensitivity [5]. In general, measures of individual characteristics have been less strongly associated with QST evidence of central pain sensitivity than are composite measures. In particular, pain distribution may be less strongly associated with QST evidence of central pain sensitivity than are other items associated with CNS function [7]. CAP-Knee contains only one (13%) pain distribution item. CAP-Knee [5] and CSI9 [16] behave as unidimensional measurement tools, and the multiple factor structure of SSS8 does not preclude it being recommended as a single summated score [12]. All of these questionnaires have shown validity and utility, and might provide useful clinical information in future studies.

Central aspects of pain are shared between people with different diagnoses, and different index joints. We have presented preliminary evidence that a CAPf may be identified in low back pain [19], as well as knee pain. CAP-Knee has some items that might be specific to knee pain; four out of eight items refer to the knee, and the widespread pain item is classified by ‘other pain below the waist’. Minor adaptations, referring to the index joint(s) rather than knee, might lead to a CAPf instrument for use across musculoskeletal conditions.

We here describe the CAP questionnaire, developed through modification of CAP-Knee, for use assessing central aspects of pain across painful musculoskeletal conditions. We evaluated acceptability, reliability and validity of CAP, in people with musculoskeletal pain, and in diagnostic subgroups with OA, back pain or FM.

## Methods

### Participants

Participants were selected from the Investigating Musculoskeletal Health and Wellbeing (IMHW) survey [20]. The IMHW is a community-based study recruiting people from the East Midlands region of the UK who were at risk of frailty, disability or musculoskeletal conditions. IMHW received favourable ethical opinion from London Central Research Ethics Committee #18/LO/0870. Recruitment to IMHW was from multiple sources detailed elsewhere [20], mostly from primary care. Recruitment to IMHW was continuous (could occur at any time) but follow-up questionnaires were dispatched in three waves, approximately annually. For validation analyses of the CAP questionnaire, data were from consecutive participants (*n* = 3579) who participated in IMHW follow up wave 2, each of whom returned a questionnaire that incorporated CAP between September 2020 and September 2021.

A nested subgroup was invited to participate in the reliability sub-study of paper questionnaires first (*n* = 168) or electronic questionnaires first (*n* = 69), then, up to 2 weeks after the questionnaires had been returned, were subsequently invited to complete electronic or paper questionnaires, respectively.

In order to examine stability or change in CAPf over time, baseline questionnaires were used from all waves. Baseline and follow up wave 1 questionnaires incorporated CAP-Knee, and waves 2 and 3 used CAP. CAP-Knee only captured data when people reported knee pain [5], and therefore only people who cited their knee as their index joint were included in this longitudinal analysis.

### CAP questionnaire

CAP was modified from the CAP-Knee questionnaire [5]. The 4 CAP-Knee items that made no reference to ‘knee’ were retained unmodified (widespread pain, depression, fatigue and anxiety) [5]. The remaining four CAP items replaced ‘knee’ with ‘joint’ (catastrophizing, cognition, sleep and neuropathic-like pain). The lead question was re-worded to ‘Please select the response that best describes how you have felt over the PAST WEEK. Joint pain may be due to pain in any of your joints, such as fingers, wrist, toes, knees, hips, etc. Please tick one box only per statement and try not to leave any statements blank’. The final paper and electronic (see Supplement 1, available at *Rheumatology* online) versions of CAP were reviewed by people with lived experience of musculoskeletal conditions.

### Demographic and clinical details

Morbidities were self-reported using tick boxes and free text. Participants reported index joint pain with the question ‘over the past 4 weeks, where was your most bothersome joint pain or aching feeling?’ Joint pain severity was recorded from 0 to 10 with the question ‘over the past 4 weeks, how intense was your average pain or average aching feeling in your most bothersome joint, where 0 is “no pain” and 10 is “pain as bad as could be”?’ Pain qualities were recorded using the full-length McGill Pain Questionnaire and its subscales for sensory, affective and evaluative pain [21]. The deciles of the English Index of Multiple Deprivation from 2019 (IMD2019) [22] were retrieved from postcodes [23].

### Statistical analysis

Most analyses were performed in the cross-sectional sample of people with joint pain in wave 2, and also in each of the three subgroups of participants with self-reported diagnoses of OA, back pain or FM. Correlation analysis identified the number of 26 body sites shaded on a pain manikin that most strongly associated with CAPf scores (derived from seven of eight CAP items, manikin excluded). This was similar to the derivation of the widespread pain item from the CAP-Knee, when widespread pain items were correlated with QST [7].

Confirmatory Factor Analysis (CFA) for one- and two-factor models were performed. Model fit was examined using indices where values close to 1.0 showed good fit (Comparative Fit Index and Tucker–Lewis Index), plus the root mean square error of approximation and standardized root mean square residual, where values close to 0 indicated better fit [24]. As each item’s data were ordinal, the diagonally weighted least squares/weighted least squares mean and variance adjusted method was used as estimator [25]. When *n* < 200, CFA was not performed [24].

Engagement with CAP was assessed by satisfaction survey, and by recording the frequency of missing data. Patterns of missingness were assessed by the association with participant. Reliability was determined in 200 participants who completed paper version and electronic questionnaires. Reliability was determined as Intraclass Correlation Coefficient (ICC). Cronbach’s alpha was derived to assess internal consistency.

Convergent validity of CAP was assessed by correlation coefficients against pain. Minimal important difference (MID) was estimated as 0.5 s.d. [26].

To inform missing items strategies, the impact of imputing missing data was modelled using data from complete questionnaires. CAP scores were calculated from all items (‘true’ CAP score), and from seven of the eight items, in separate models in which the same item was removed from all questionnaires. CAP scores were imputed with the average (rounded integer) of the remaining seven items. Scores were also examined when two items were removed, and imputed using the mean integer. When two items were removed, they were selected to represent the most likely combinations to skew or bias the imputed CAP data. Median [interquartile range (IQR)] and Bland–Altman plots were derived to compare true CAP scores and imputed data.

Heterogeneity between time points was assessed by non-parametric Friedman’s test. This tested whether the difference in CAP between longitudinal time points for individuals differed from zero, indicative of a variable trait. Changes between time points for each individual were classified as being greater than the minimum important difference for CAP, numerical rating scale (NRS) for pain and McGill Pain Questionnaire total score.

Statistical analyses used R with lavaan, ltm and irr packages. Heterogeneity between timepoints was assessed using SPSS version 26 (IBM, Chicago, MI, USA).

## Results

A total of 4130 people had returned IMHW questionnaires in IMHW follow up wave 2 at the time of the study (see Supplement 2, available at *Rheumatology* online), and the study population that reported joint pain (*n* = 3579) is shown in Table 1. People with joint pain reported diagnoses of OA (*n* = 1158), back pain (*n* = 1292) or FM (*n* = 177). Median pain scores were highest in the FM subgroup.

**Table 1.** Description of the people reporting joint pain

	Participants with joint pain	OA	Back pain	FM	Reliability and feedback subgroup	Longitudinal analysis group
Variable	Median (IQR) or %	Median (IQR) or %	Median (IQR) or %	Median (IQR) or %	Median (IQR) or %	Median (IQR) or %
*N*	3579	1153	1292	177	200	2155
Age, years	71 (66, 77)	72 (66, 77)	71 (65, 76)	67 (55, 74)	70 (65, 74)	71 (63, 77)
Female, %	60	70	64	86	56	57
White race, %	97	98	97	95	100	94
BMI, kg/m^2^	27.1 (24.2, 30.6)	27.6 (24.8, 31.4)	27.4 (24.7, 31.0)	28.5 (26.0, 32.9)	26.9 (24.2, 30.2)	28.2 (25.1, 32.0)
Area deprivation, IMD2019 decile	7 (5, 9)	7 (5, 9)	7 (5, 9)	6 (4, 9)	8 (6, 10)	7 (5, 9)
Joint pain severity, 0–10	6 (4, 7)	6 (5, 8)	7 (5, 8)	7 (6, 8)	5 (4, 7)	6 (5, 8)
McGill total, 0–78	12 (7, 21)	15 (9, 25)	16 (9, 26)	22 (14, 36)	12 (6, 18)	13 (7, 23)
McGill sensory, 0–42	8 (4, 14)	10 (6, 15)	10 (6, 16)	13 (9, 20)	8 (4, 13)	9 (5, 15)
McGill affective, 0–14	0 (0, 2)	1 (0, 2)	1 (0, 3)	2 (0, 6)	0 (0, 1)	1 (0, 2)
McGill evaluative, 0–5	2 (1, 3)	2 (1, 4)	2 (1, 4)	3 (1, 4)	2 (1, 3)	2 (0, 3)

Description of the people reporting joint pain. IMD2019 English Area Deprivation decile ranges from 1 (worst) to 10 (least). IQR: interquartile range; IMD2019: English Index of Multiple Deprivation from 2019.

Participants reported high satisfaction with the CAP questionnaire for both paper and electronic versions, with 92% indicating that it was easy to follow and 99% that they would be happy to complete the questionnaire again. Nine percent of participants (330/3579) did not complete all CAP items; 4% (147/3579) of people omitted two or more items; and 5% (183/3579) omitted one item. Neuropathic-like pain was the most frequently omitted item (*n* = 195, 5%). Missing items were associated with slightly lower McGill questionnaire sensory pain scores [median (IQR), 8 (4, 12) *vs* 8 (5, 14), *P* = 0.006] and older age [median (IQR), 74 (69, 80) *vs* 70 (66, 76) years, *P* < 0.001], and were not significantly associated with joint pain severity NRS [6 (4, 8) *vs* 6 (4, 7), *P* = 0.485], sex (9% female *vs* 10% male, *P* = 0.248) or social deprivation rank [IMD2019 decile; 8 (6, 9) *vs* 7 (5, 9), *P* = 0.127].

**Figure 1.**
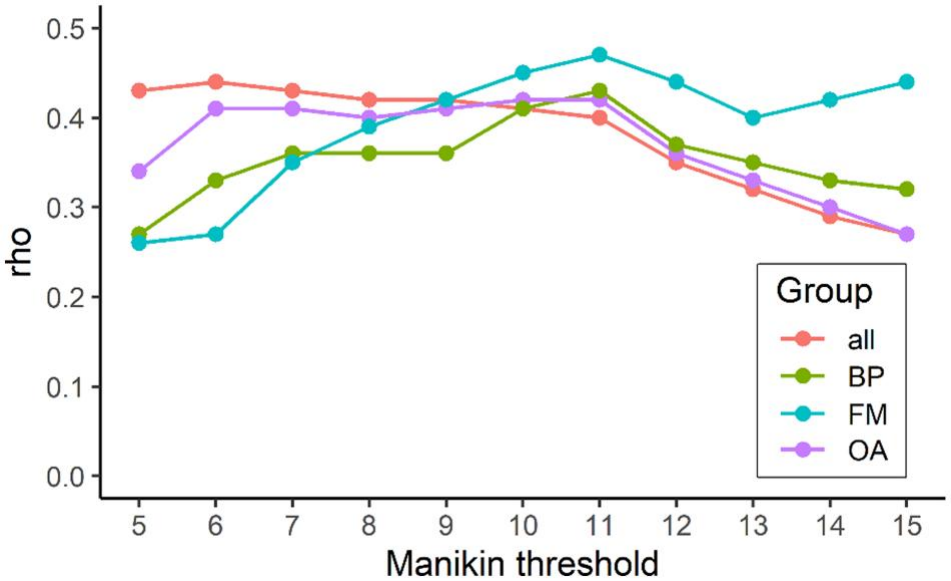
Comparisons of correlation coefficients between different manikin region counts and CAPf. CAPf was estimated using a modified CAP derived by summation of seven out of eight items, excluding manikin. Spearman’s rho values represent associations between seven manikin widespread pain criteria (using thresholds ranging from 5 to 15 out of 26 regions) and CAPf (derived from the remaining seven items). Rho values converged across diagnostic groups with a manikin threshold of 10 (10 or more sites assessed as widespread pain) (rho = 0.41 for complete sample, 0.42 for OA, 0.41 for back pain and 0.45 for FM). Subsequently, a threshold of ≥10/26 painful sites on the manikin was selected to score people as exhibiting widespread pain (score = 2), and ≤9/26 painful sites to score as not widespread pain (score = 0) for calculating CAPf. CAP: Central Aspects of Pain; CAPf: Central Aspects of Pain factor; all: complete sample; BP: back pain

A common criterion across diagnoses for widespread pain was sought. More widespread pain, defined using a range of thresholds from 5 to 15 out of 26 painful sites on the body manikin, was significantly associated with modified CAP scores derived from the remaining seven items (Fig. 1). Ten or more out of 26 painful sites provided a convergent threshold across diagnostic groups.

**Figure 2.**
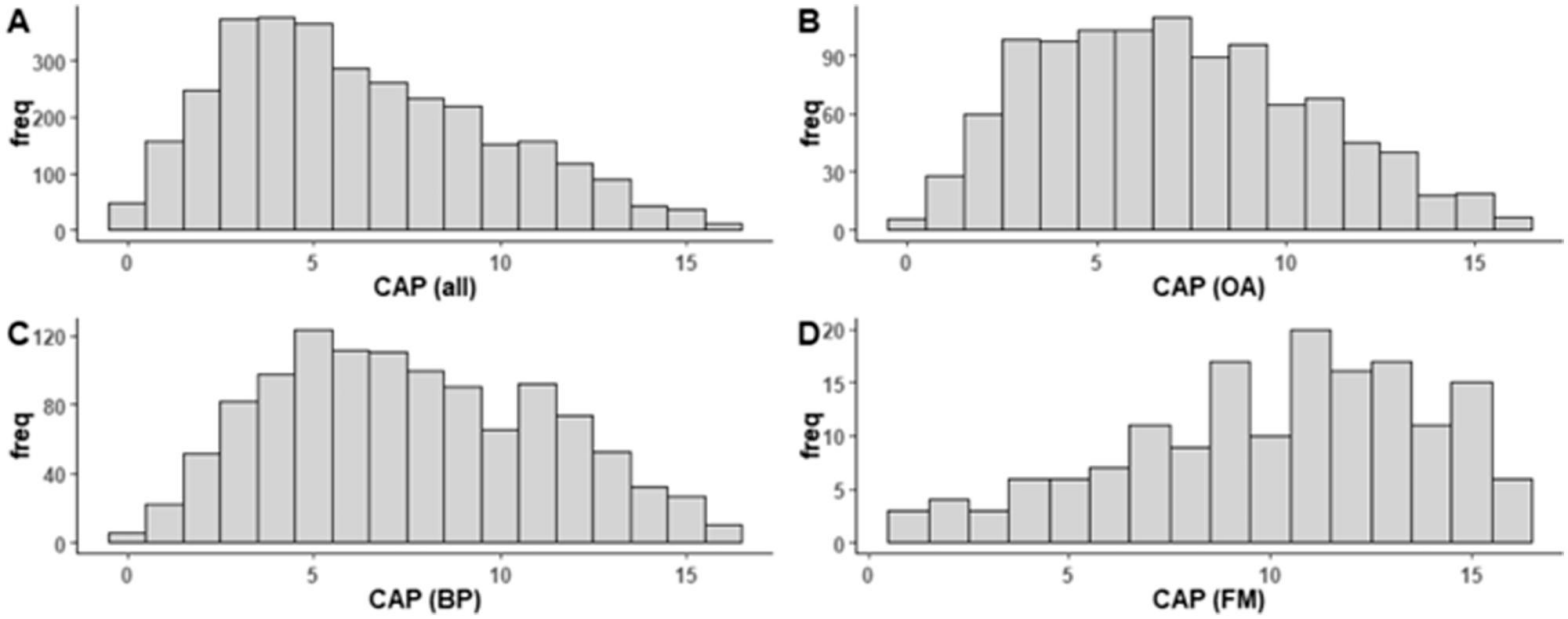
Distribution of CAP scores. Distributions of CAP scores for whole population. CAP score distributions were positively skewed in the subgroups with OA or back pain, and negatively skewed in people with FM. (**A**) All [skewness = 0.52 (95% CI 0.46, 0.58), kurtosis = 2.53 (95% CI 2.43, 2.65)]. (**B**) OA [skewness = 0.52 (95% CI 0.46, 0.57), kurtosis = 2.39 (95% CI 2.26, 2.54)]. (**C**) Lower back pain [skewness = 0.24 (95% CI 0.15, 0.32), kurtosis = 2.22 (95% CI 2.11, 2.34)]. (**D**) FM [skewnesss = –0.51 (95% CI –0.77, –0.27), kurtosis = 2.51 (95% CI 2.16, 3.09)]. CAP: Central Aspects of Pain; all: complete sample; BP: back pain

CAP score distribution was unimodal within those who reported joint pain, and included all possible scores (0–16, Fig. 2). Floor [*n* = 50 (1%) when CAP = 0] and ceiling [*n* = 15 (0.4%) when CAP = 16] effects were not substantial (Fig. 2). Median (IQR) baseline CAP scores were: IMHW, 6 (3, 9); OA 7 (4, 9); back pain, 7 (5, 11); and FM, 11 (8, 13). The 0.5 s.d. for baseline CAP scores was 1.8, to give an estimate for MID of 2 points [26].

CFA indicated that each item could contribute to a single factor model with good fit. Data also fitted well to a two-factor model which showed very high covariance (0.87–0.90) between the two factors (Table 2). Items were therefore summated for a total score. High reliability was demonstrated between paper and electronic CAP questionnaires, with ICC = 0.89 (95% CI 0.84–0.92) and Cronbach’s alpha for CAP items = 0.79 (95% CI 0.78–0.80).

**Table 2.** Factor loadings for confirmatory factor analysis

All data								
	One factor					Two factors		
CFI	0.980					0.991		
TLI	0.972					0.987		
RMSEA (95% CI)	0.065	(0.058 to 0.071)				0.044	(0.037 to 0.051)	
SRMR	0.046					0.033		
Covariance (s.e.) between factors	Not applicable					0.866	(0.012)	

	Standardized estimate	s.e.	*P*		Factor	Standardized estimate	s.e.	*P*

Neuropathic-like	0.61	0.018	<0.001		Factor 1	0.64	0.019	<0.001
Fatigue	0.67	0.014	<0.001		Factor 1	0.71	0.014	<0.001
Depression	0.42	0.018	<0.001		Factor 1	0.44	0.019	<0.001
Anxiety	0.70	0.017	<0.001		Factor 1	0.74	0.018	<0.001
Widespread pain	0.65	0.021	<0.001		Factor 1	0.68	0.022	<0.001
Sleep	0.71	0.010	<0.001		Factor 2	0.72	0.013	<0.001
Catastrophizing	0.80	0.010	<0.001		Factor 2	0.81	0.010	<0.001
Cognition	0.87	0.009	<0.001		Factor 2	0.90	0.009	<0.001

**OA**								

	One factor					Two factors		
CFI	0.981					0.991		
TLI	0.973					0.987		
RMSEA (95% CI)	0.061	(0.050 to 0.071)				0.043	(0.030 to 0.056)	
SRMR	0.046					0.036		
Covariance (s.e.) between factors	Not applicable					0.871	(0.021)	

	Standardized estimate	s.e.	*P*		Factor	Standardized estimate	s.e.	*P*

Neuropathic-like	0.57	0.032	<0.001		Factor 1	0.60	0.033	<0.001
Fatigue	0.69	0.023	<0.001		Factor 1	0.73	0.024	<0.001
Depression	0.48	0.029	<0.001		Factor 1	0.51	0.030	<0.001
Anxiety	0.67	0.031	<0.001		Factor 1	0.70	0.032	<0.001
Widespread pain	0.63	0.034	<0.001		Factor 1	0.66	0.035	<0.001
Sleep	0.69	0.023	<0.001		Factor 2	0.71	0.024	<0.001
Catastrophizing	0.78	0.019	<0.001		Factor 2	0.80	0.019	<0.001
Cognition	0.86	0.017	<0.001		Factor 2	0.88	0.018	<0.001

**Back pain**								

	One factor					Two factors		
CFI	0.985					0.990		
TLI	0.978					0.985		
RMSEA (95% CI)	0.056	(0.044 to 0.068)				0.046	(0.034 to 0.058)	
SRMR	0.042					0.036		
Covariance (s.e.) between factors	Not applicable					0.903	(0.020)	

	Standardized estimate	s.e.	*P*		Factor	Standardized estimate	s.e.	*P*

Neuropathic-like	0.61	0.029	<0.001		Factor 1	0.63	0.030	<0.001
Fatigue	0.67	0.024	<0.001		Factor 1	0.69	0.025	<0.001
Depression	0.55	0.027	<0.001		Factor 1	0.57	0.028	<0.001
Anxiety	0.69	0.026	<0.001		Factor 1	0.71	0.027	<0.001
Widespread pain	0.61	0.033	<0.001		Factor 1	0.63	0.033	<0.001
Sleep	0.66	0.023	<0.001		Factor 2	0.67	0.024	<0.001
Catastrophizing	0.76	0.019	<0.001		Factor 2	0.77	0.019	<0.001
Cognition	0.86	0.015	<0.001		Factor 2	0.88	0.016	<0.001

Comparison of CFA standardized loadings for one- and two-factor models of CAP. The two-factor model consisted of items loading onto either factor 1 (items for neuropathic-like, fatigue, cognition, depression and anxiety) or loading onto factor 2 (items for cognition, sleep and catastrophizing). Populations consisted of all participants with joint pain and CAP data (*n* = 3177), OA (*n* = 1052) or back pain (*n* = 1151). The number of people who self-reported FM (*n* = 177) was insufficient for analysis. CFI: Comparative Fit Index; TLI: Tucker–Lewis Index; RMSEA: root mean square error of approximation; SRMR: standardized root mean square residual; CAP: Central Aspects of Pain.

**Figure 3.**
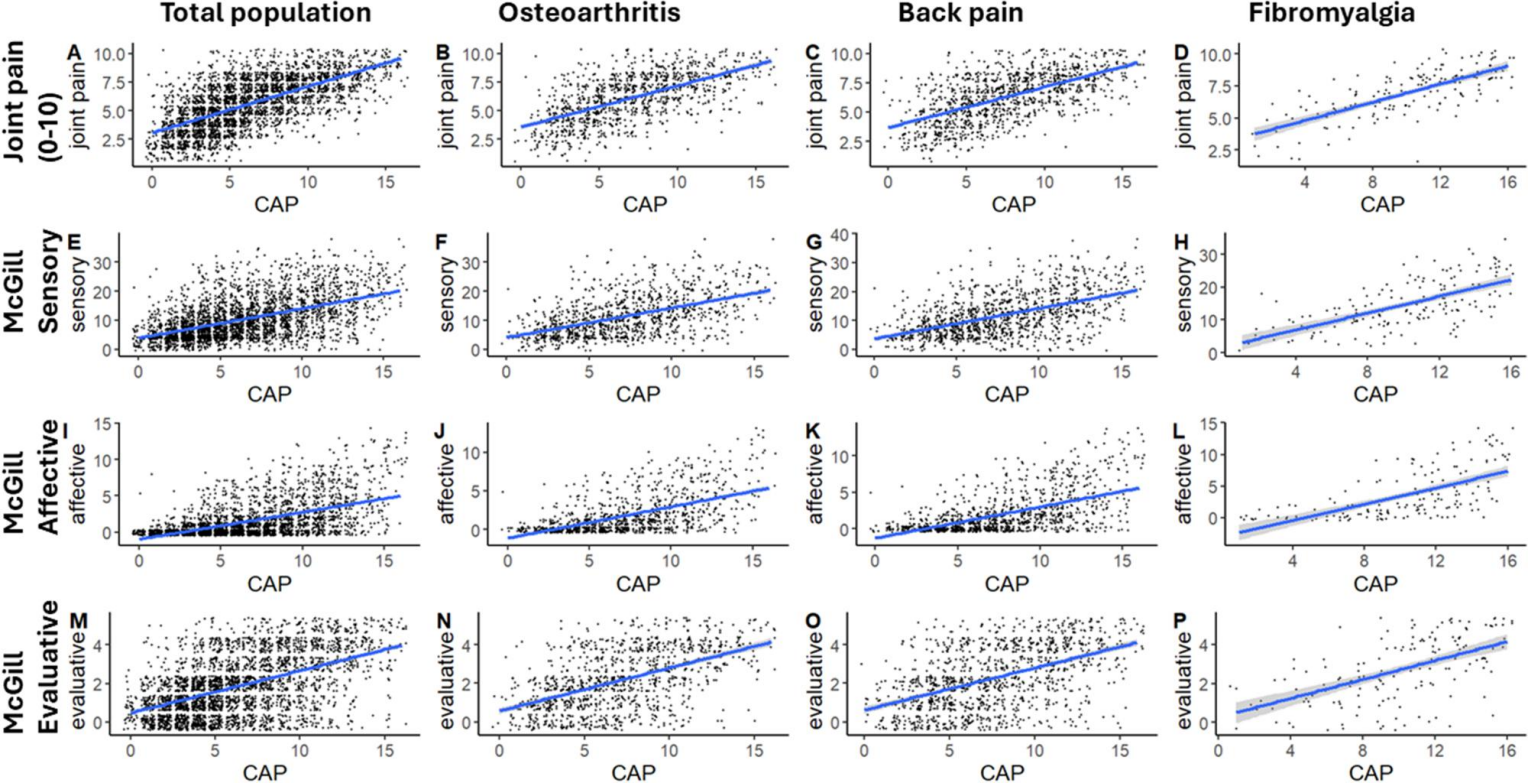
Convergent validation of CAP questionnaire by correlation with pain scales. Scatterplots of CAP *vs* pain scales. (**A**, **E**, **I**, **M**) Total study population; CAP *vs* joint pain, McGill sensory, McGill affective, McGill evaluative scales. (**B**, **F**, **J**, **N**) OA population; CAP *vs* joint pain, McGill sensory, McGill affective, McGill evaluative scales. (**C**, **G**, **K**, **O**) Back pain population; CAP *vs* joint pain, McGill sensory, McGill affective, McGill evaluative scales. (**D**, **H**, **L**, **P**) FM population; CAP *vs* joint pain, McGill sensory, McGill affective, McGill evaluative scales. Linear line of best fit (95% CI) shown for each comparison. Correlation coefficients (p) for each panel were (A) 0.66 (<0.001), (B) 0.62 (<0.001), (C) 0.62 (<0.001), (D) 0.72 (<0.001), (E) 0.52 (<0.001), (F) 0.52 (<0.001), (G) 0.52 (<0.001), (H) 0.65 (<0.001), (I) 0.57 (<0.001), (J) 0.58 (<0.001), (K) 0.58 (<0.001), (L) 0.63 (<0.001), (M) 0.52 (<0.001), (N) 0.52 (<0.001), (O) 0.50 (<0.001), (P) 0.57 (<0.001). CAP: Central Aspects of Pain

CAP was positively associated with NRS joint pain (r = 0.66, *P* < 0.0001), McGill sensory scale (r = 0.52, *P* < 0.0001) and other measures of pain quality (Fig. 3). For people with OA, back pain or FM, the correlation coefficients with NRS joint pain were 0.62, 0.62 and 0.72, respectively; and with McGill sensory subscale were 0.51, 0.52 and 0.65, respectively.

**Figure 4.**
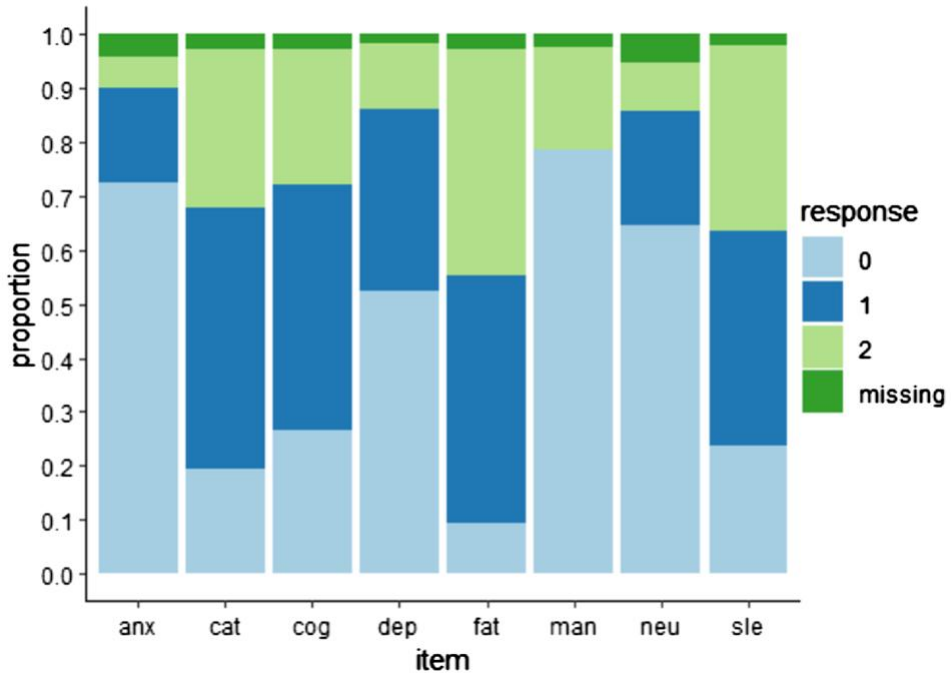
Distribution of item responses. Frequency of item responses, are represented as a proportion of total, including missing data. Responses 0 = never, 1 = sometimes, 2 = often and always. Anx: anxiety item; cat: catastrophizing item; cog: cognition item; dep: depression item; fat: fatigue item; man: widespread pain (manikin) item; neu: neuropathic-like pain item; sle: sleep item

The distribution of participants’ responses to CAP items is shown in Fig. 4. Complete CAP data had items sequentially removed and imputed. Median imputed CAP was identical to the original when a single item was removed, except with removal of the fatigue item (median imputed CAP 1 point lower, Supplements 3 and 4, available at *Rheumatology* online). Where two items were removed, imputed scores often deviated from true scores by 2 points (see Supplement 5, available at *Rheumatology* online).

CAPf stability over time was investigated in participants with knee pain. Supplement 2, available at *Rheumatology* online, shows participant numbers across data collection waves. Median (IQR) CAP scores were similar between waves [baseline, 8 (5, 11), *n* = 2137; wave 1 (1 year), 8 (5, 11), *n* = 766; wave 2 (2 years), 9 (6, 12), *n* = 681; and wave 3 (4 years), 9 (7, 12), *n* = 533]. The median (IQR) changes between time points within each participant were; baseline to wave 1, 0 (–2, 2), *n* = 761; wave 1–2, 0 (–1, 2), *n* = 404; and wave 2–3, 0 (–1, 2), *n* = 275. Significant changes in CAP were found within individuals over time (Friedman test; χ^2^ = 210.131, 3 df, *P* < 0.001, *n* = 461). The frequency of changes in CAP that were greater than the minimum important difference are shown in Supplements 6 and 7, available at *Rheumatology* online, with >50% displaying an important change at each measurement.

## Discussion

We here report the 8-item CAP questionnaire, derived from the CAP-Knee questionnaire, and show that its measurement is consistent with CAPf across diverse, chronically painful musculoskeletal conditions. CAP was acceptable to participants, in both paper and electronic versions, with low item missingness. A threshold of ≥10/26 painful sites shaded on the body manikin best assessed widespread pain. CAP score distributions were unimodal with a calculated MID of 2 points. Single missing items can be imputed from an individual’s score for the remaining seven items. CAP scores associated both with pain severity and pain quality. CAP scores were highly repeatable over a period of weeks and showed little variability over 3 years at the group level, but displayed significant and clinically important temporal heterogeneity within individuals. We here extend our previous findings in people with knee pain, to show that CAPf displays generalizable validity in people with pain at single or multiple index joints.

Chronic pain is both a sensory and emotional experience, resulting from the integration of mechanisms within the peripheral and central nervous systems. Our current findings are consistent with previous evidence that self-reported characteristics of neuropathic-like pain quality, pain distribution beyond a site of tissue injury, fatigue, cognitive difficulty, catastrophizing, anxiety, sleep disturbance and depression, each is associated with pain severity in people with musculoskeletal pain [7, 27]. Furthermore, each of these characteristics has been associated with reduced PPTs distal to chronically painful knees [7, 28–30]. Reduced pain detection thresholds distant or distal to a site of pathology may indicate central sensitization [31], and central pain processing might influence how musculoskeletal pain is experienced or reported.

Widespread pain distribution is associated with greater pain severity, and evidence of central pain sensitivity [32, 33]. Several methods have been used to classify or measure pain distribution. The ACR developed the Widespread Pain Index (WPI), which became a classification criterion for FM [34, 35]. Other authors have used number of body sites on a pain manikin [19]. CAP-Knee selected ‘other pain below the waist’ as being most closely associated with low PPT distal to an index knee [5]. In people with knee pain, WPI displayed only weak correlation with PPT. Our approach to the widespread pain item was designed to maximize CAP internal consistency. Across different diagnoses a threshold of ≥10/26 painful sites provided a consistently high correlation with CAPf, despite these conditions being characterized by different pain distributions.

We minimized changes to CAP-Knee when developing CAP in order to build on our previous research that maximized associations with PPT, and that was comprehensible to people with pain [5]. Where the index site is knee, CAP performs as expected, and our current findings confirm it is consistent with a unitary scale. Ninety-one percent of returned questionnaires included responses to all eight items. Missing CAP items were similar across sex and socioeconomic strata, suggesting broad acceptability. Small but statistically significant higher item missingness was found with older participants, and those with lower McGill sensory pain scores. It is possible that older people with less severe pain might have difficulty assigning values to pain-associated characteristics, especially if pain were sporadic or not viewed as a major problem. Our data imputation findings lead us to propose that a single missing item can be replaced by rounded mean integer imputation from the remaining seven items. Two or more items were missing within a single returned questionnaire in 4% of responses, and when modelled data manipulation from complete responses resulted in divergence between true and imputed CAP scores frequently greater than the MID (2 points). Caution therefore should be exercised in producing CAP scores if fewer than seven items are completed. Detailed scoring instructions for CAP are given in Supplement 1, available at *Rheumatology* online.

CAP scores displayed convergent validity through their associations with pain severity and quality, including the sensory subscale of the McGill Pain Questionnaire. These associations were observed across diagnostic groups, and were strongest in those with FM and weakest in OA, consistent with previous evidence that central aspects of pain are more predominant in FM than in OA [36].

Our data fitted well to both one- and two-factor models, with very high co-correlation in all analyses between factors in the two-factor model. Both models appear consistent with a scale derived by summation of all eight items, which might measure an overarching aspect of pain. Results of the two-factor CFA would be consistent with subscales within CAP and would also indicate a reduced comparability between CAP and CAP-Knee. However, further research would need to determine whether a two-factor structure were replicated in other populations, and to investigate the biological meaning of a subscore based on cognition, catastrophizing and sleep items. We previously referred to a unitary factor underlying CAP-Knee as ‘Central Mechanisms Trait’. However, we here show that CAP scores, although stable over periods of weeks, show frequent changes over 3 years. The factor measured by CAP and CAP-Knee therefore appears to be a changeable state, rather than an intrinsic trait in people with knee pain. That CAP-Knee scores predicted pain outcomes suggests a causal mechanistic interpretation [8], but it is also possible that scores can represent consequences of pain. We therefore here refer to a CAPf in order to avoid mechanistic overinterpretation of our findings. CAP is not a direct measure of central sensitization or neuropathy. Indeed, central sensitization may be a heterogeneous condition that results from multiple discrete mechanisms within the brain and spinal cord. Central sensitization in humans cannot be measured by any single ‘gold standard’ tool, and further research should define relationships between CAP and discrete aspects of central pain sensitivity.

Recent attention has focused upon nociplastic pain—‘pain that arises from altered nociception despite no clear evidence of actual or threatened tissue damage causing the activation of peripheral nociceptors or evidence for disease or lesion of the somatosensory system causing the pain’ [1]. Nociplastic pain can occur alongside neuropathy or musculoskeletal pathology. Classification of nociplastic pain requires evidence of pain hypersensitivity, and symptoms of sleep disturbance, fatigue and cognitive problems [37]. We show that these symptoms are associated with CAPf, as were lower pressure pain detection thresholds [7], indicative of pain hypersensitivity. CAPf might therefore be an index of nociplasticity. However, multiple mechanisms might contribute to nociplasticity, and CAP might measure each of these only partially. Variations in CAPf over time within individuals suggests the potential to be modifiable and therefore could be developed and validated as a potential target for treatment. Research is also underway examining alternative self-report questionnaires, such as CSI9, SSS8 and the Keele STarT MSK Tool, which might also represent treatment targets or predictive tools. Studies comparing different instruments in different populations with chronic pain are warranted [38].

Our study has several strengths, but also several limitations. This study did not directly demonstrate the ability of CAP to measure or classify pain mechanisms. CAP displayed convergent validity against measures of pain, but use alongside other indices of nociplastic pain mechanisms, such as QST, might further improve its clinical value. Future work might assess whether CAP can show utility over and above other instruments or QST modalities. The study population was almost entirely white British with or at risk of musculoskeletal pain or frailty. We did not undertake assessments to confirm self-reported diagnoses. We did not investigate all musculoskeletal conditions. The generalizability of our findings requires further investigation. We confirmed factor structures for subgroups with OA or low back pain, but our FM subgroup was of insufficient size. However, consistency of our findings across three different diagnostic groups, and in the total study population, strongly implies that CAP could show similar properties in other conditions. CAPf was associated with pain severity, supporting its identification as a pain-related characteristic. However, CAP scores might be confounded by nociceptive or neuropathic pain severity. Pain severity is not readily adjusted out of statistical models without the possibility of introducing bias. CAP was developed to explore mechanistic aspects of musculoskeletal pain that might have prognostic or predictive value. As such it might complement, rather than replace, existing questionnaires that have been designed to address patient concerns about their pain, its impact on their lives or their overall well-being. Factors additional to central pain sensitivity can influence pain prognosis, and CAP might complement prognostic tools such as the Keele STarT MSK Tool [17, 18] by identifying possible contribution to prognosis from central aspects of pain.

In conclusion, we report the CAP questionnaire as a possible measurement tool for nociplastic pain. Future research should test the mechanistic underpinning of CAP, and its ability to predict future pain and responses to treatment.

## Supplementary Material

keae342_Supplementary_Data

## Data Availability

Data requests can be addressed to D.A.W., e-mail: David.walsh@nottingham.ac.uk.
