# Supplementary material for: Validation of a questionnaire for central nervous system aspects of joint pain: the CAP questionnaire
Source: Rheumatology (Oxford). 2024 Jun 18;63(12):3306–14. doi: 10.1093/rheumatology/keae342 (PMC11637516; doi:10.1093/rheumatology/keae342)
Supplement: keae342_Supplementary_Data [file keae342_supplementary_data.docx]

**Supplement 1: Paper and electronic versions of the CAP questionnaire with scoring guide**

Please select the response that best describes how you have felt over the PAST *WEEK*. Joint pain may be due to pain in any of your joints, such as fingers, wrist, toes, knees, hips, etc.

Please tick one box only per statement and try not to leave any statements blank.

|  |  | never | sometimes | often | always |
| --- | --- | --- | --- | --- | --- |
| 1 | Cold or heat (eg. bath water) on my joint was painful |  |  |  |  |
| 2 | I generally felt tired |  |  |  |  |
| 3 | My joint pain stopped me concentrating on what I was doing |  |  |  |  |
| 4 | I kept thinking about how much my joint hurts |  |  |  |  |
| 5 | In general, I got sudden feelings of panic |  |  |  |  |
| 6 | Joint pain affected my sleep |  |  |  |  |
| 7 | I generally still enjoyed the things I used to enjoy |  |  |  |  |
| 8 | This next question is about pain you may have had in any part of your body. Please shade in the diagram below, to indicate where you have suffered any pain for most days in the last ***4 WEEKS***. By pain we also mean aching and/or discomfort. Please do not include pain due to feverish illness such as flu.  R  L  R  L  R  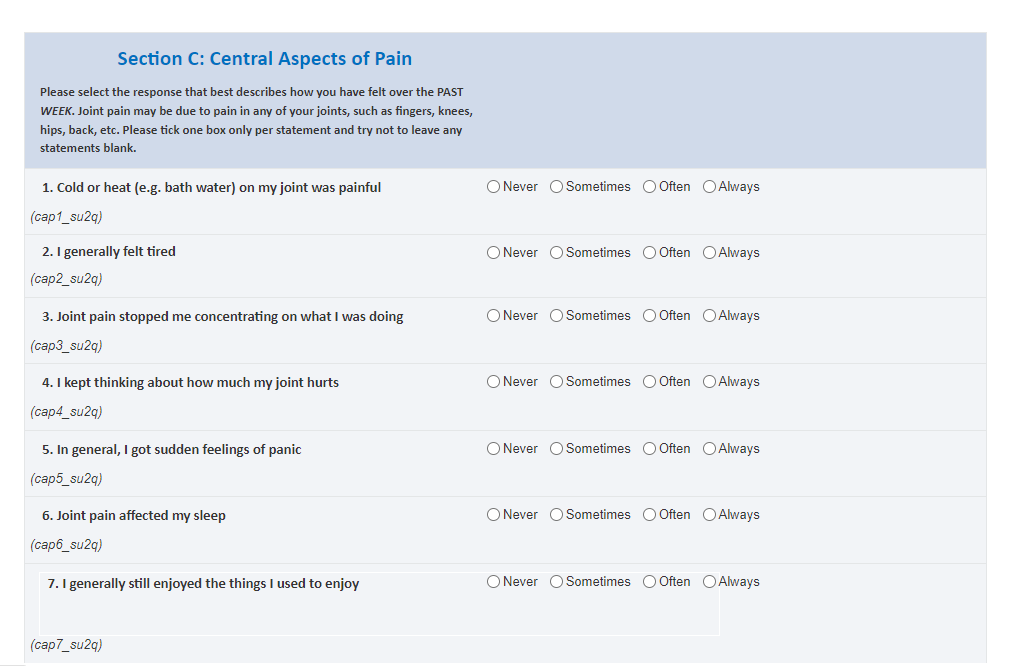 | | | | |


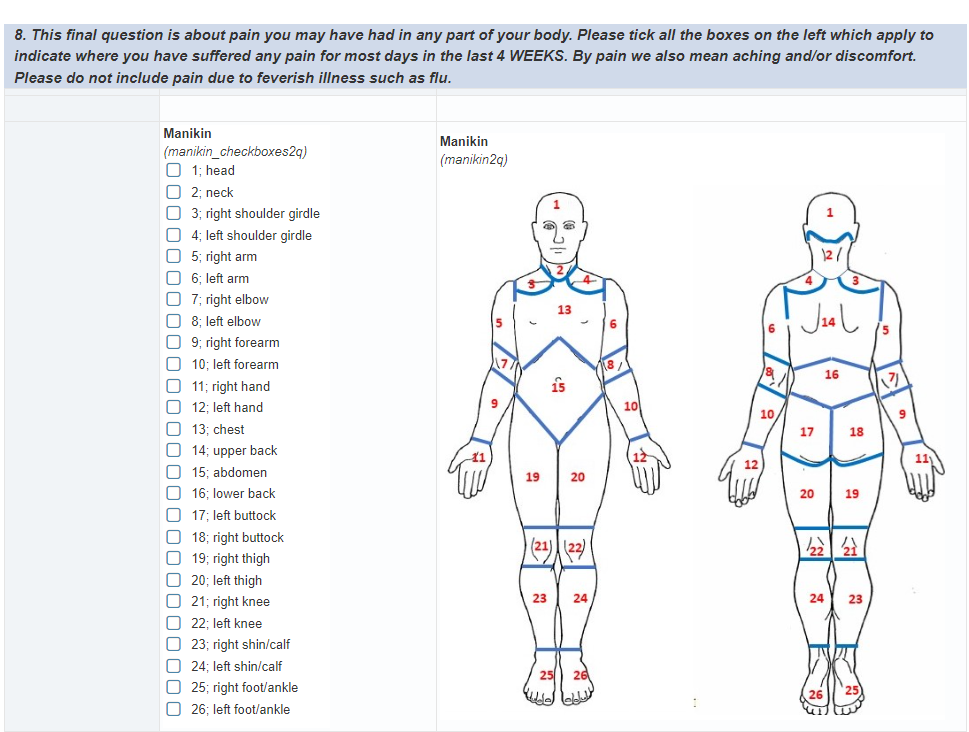


**CAP scoring guide**

**Never=0, Sometimes=1, Often=2, Always=2**

1. Cold or heat (eg. bath water) on my joint was painful

2. I generally felt tired

3. My joint pain stopped me concentrating on what I was doing

4. I kept thinking about how much my joint hurts

5. In general, I got sudden feelings of panic

6. Joint pain affected my sleep

**Never=2, Sometimes=1, Often=0, Always=0**

7. I generally still enjoyed the things I used to enjoy

**Body pain manikin scoring guide.**

Count number of regions with shading or markings from the participant

0 to 9 regions = 0

10 or greater regions = 2

When pain is recorded as both front and back of a particular region, it is counted as 1 region (and not 2).


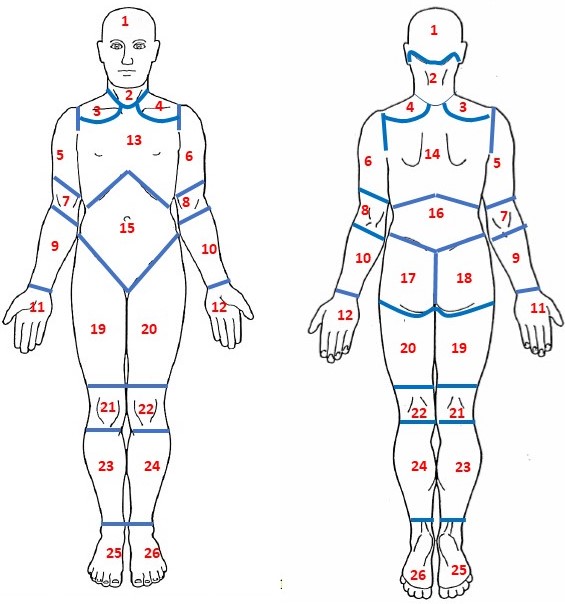


Supplement 2: CONSORT flow diagram of participant pathway


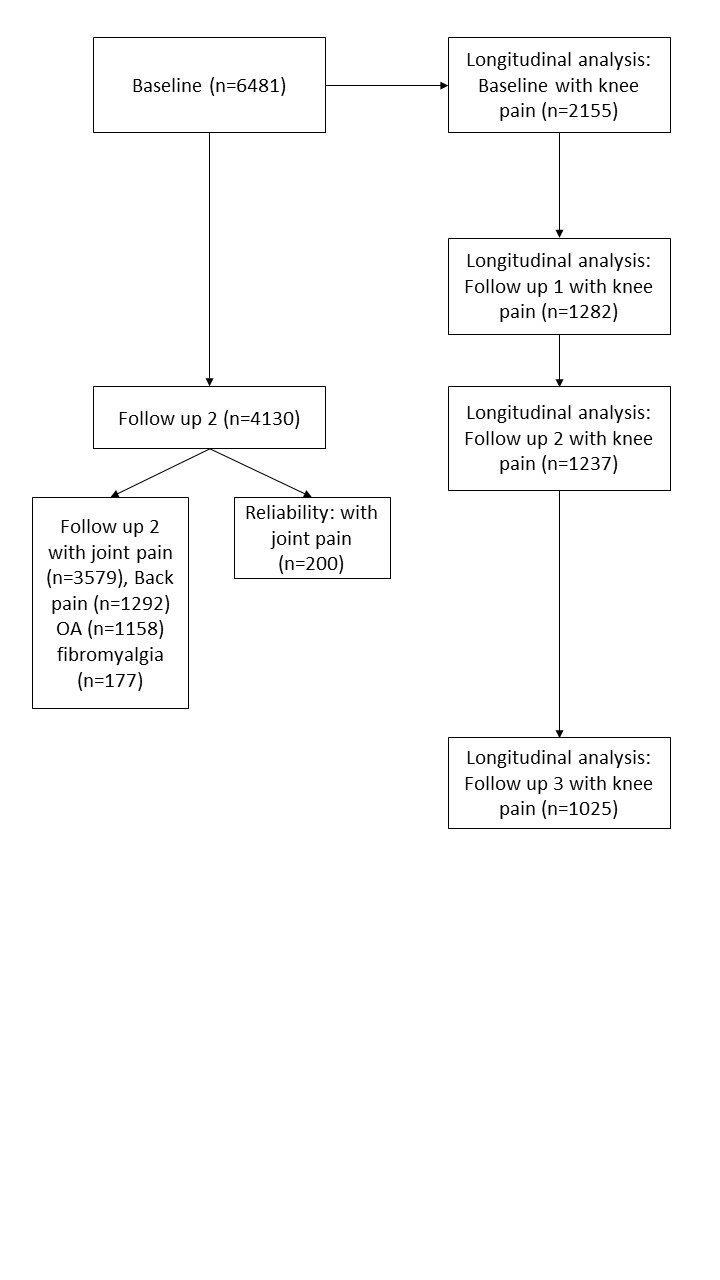


**Supplement 3: box and whiskers plot of complete CAP score compared to sequential imputation of a single missing item**
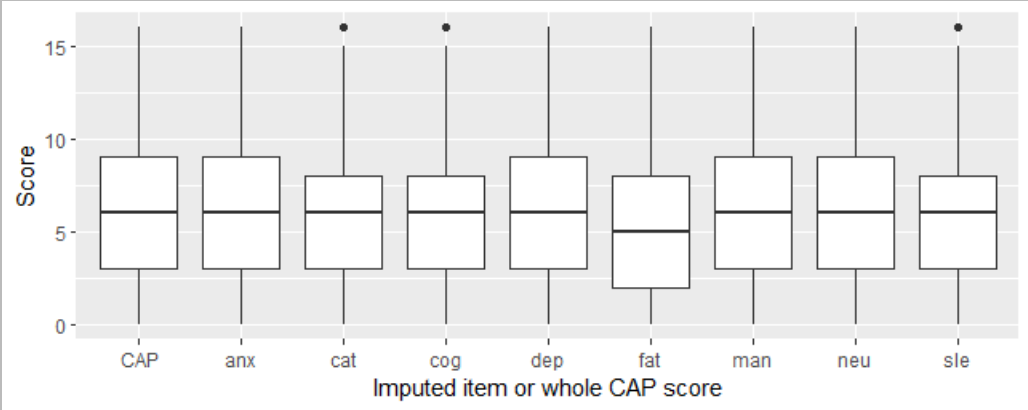


CAP= whole CAP score. Scores imputed from a single missing item are shown.

**Supplement 4: Comparison between CAP and scores made with 1 missing value that was mean imputed**


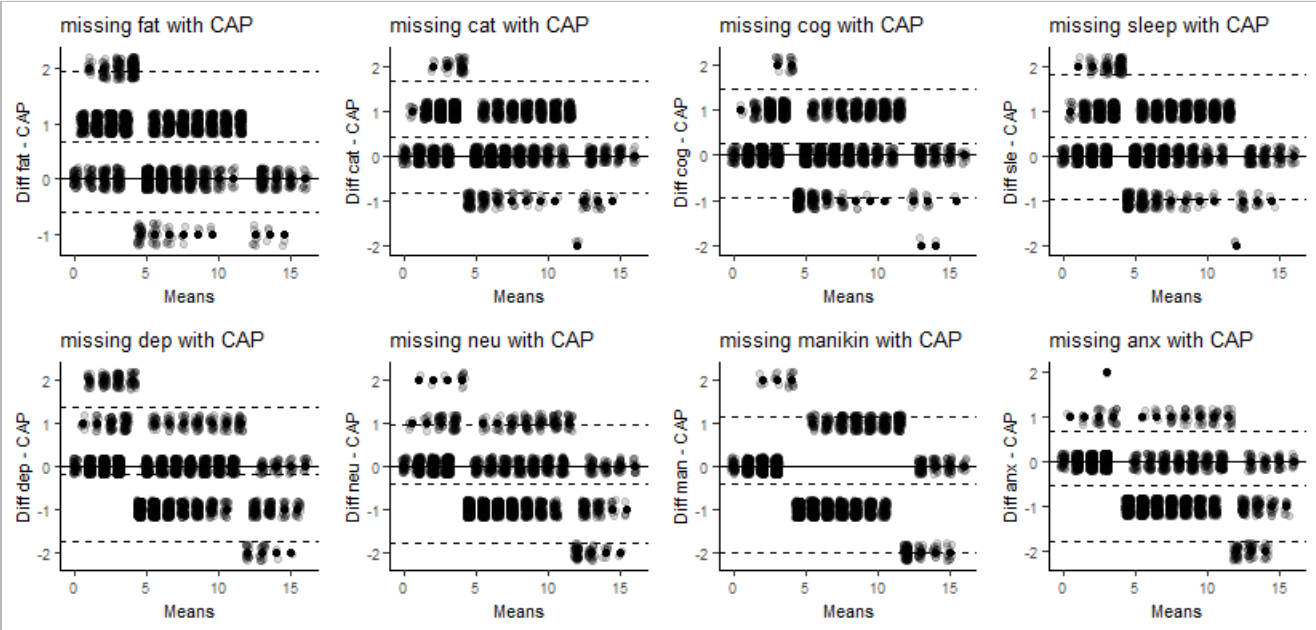


Bland Altman plots of CAP (y axis) vs imputed CAP with one value removed systematically. On the y-axis the difference of zero is shown with a solid line. Mean difference and upper/lower limits of agreement are shown as dotted lines. Abbreviations: CAP-Central Aspects of Pain score derived from remaining items; fat – fatigue item; cat – catastrophising item; cog – cognition item, sleep – sleep item; dep – depression item, neu – neuropathic-like pain item; manikin – manikin item for widespread pain, anx – anxiety item.

Supplement 5: CAP scores derived from imputation of 2 missing items using the item response mean


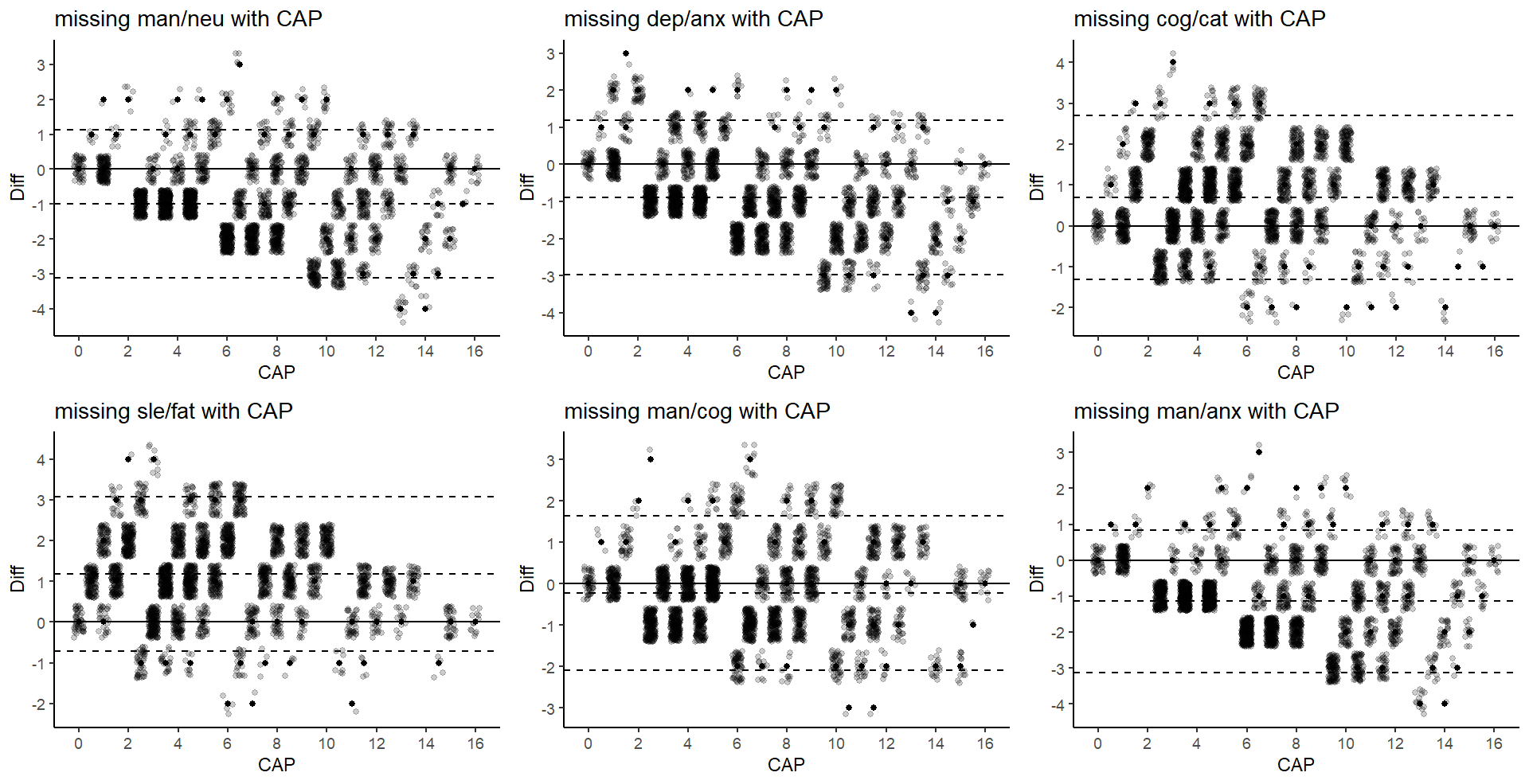


Bland Altman plots of “true” CAP (y-axis) and the distribution of differences between the derived CAP with 2 missing items and CAP. Bland Altman plots of CAP (y axis) vs imputed CAP with one value removed systematically. On the y-axis the difference of zero is shown with a solid line. Mean difference and upper/lower limits of agreement are shown as dotted lines. In the study population, missing numbers for each of these combinations were: man/neu 17; dep/anx 49; cog/cat 70; sle/fat 44; man/cog 15; man/anx 17. Abbreviations: CAP-Central Aspects of Pain score derived from remaining items; fat – fatigue item; cat – catastrophising item; cog – cognition item, sleep – sleep item; dep – depression item, neu – neuropathic-like pain item; manikin – manikin item for widespread pain, anx – anxiety item.

**Supplement 6:**

Distribution of participants (knee pain only) with deteriorating, stable or improved questionnaire scores between assessments

| **Variable** | **Change over time** | **N** | **Worse** | **No Change** | **Better** |
| --- | --- | --- | --- | --- | --- |
| **CAP score**  **(MID =2)** | Baseline/FU1 | 761 | 202 (27%) | 329 (43%) | 230 (30%) |
|  | FU1/FU2 | 404 | 141 (35%) | 185 (46%) | 78 (19%) |
|  | FU2/FU3 | 275 | 75 (27%) | 133 (48%) | 67 (24%) |
| **NRS Pain Intensity**  **(MID =2)** | Baseline/FU1 | 726 | 116 (16%) | 454 (63%) | 156 (21%) |
|  | FU1/FU2 | 400 | 74 (19%) | 264 (66%) | 62 (16%) |
|  | FU2/FU3 | 271 | 50 (18%) | 174 (64%) | 47 (17%) |
| **McGill total score**  **(MID =7)** | Baseline/FU1 | 759 | 142 (19%) | 425 (56%) | 192 (25%) |
|  | FU1/FU2 | 404 | 95 (24%) | 241 (60%) | 68 (17%) |
|  | FU2/FU3 | 275 | 4 (1%) | 135 (49%) | 136 (49%) |

CAP – Central Aspects of Pain in the knee; MCID= Minimal clinically important difference (0.5* baseline standard deviation); NRS – Numerical rating scale for Pain Intensity; FU1 – Follow-up year 1; FU2 – Follow-up year 2; FU3 – Follow-up year 3; MID – minimally important difference.

**Supplement 7:** Distribution of participants (knee pain only) with deteriorating, stable or improved questionnaire scores between assessments


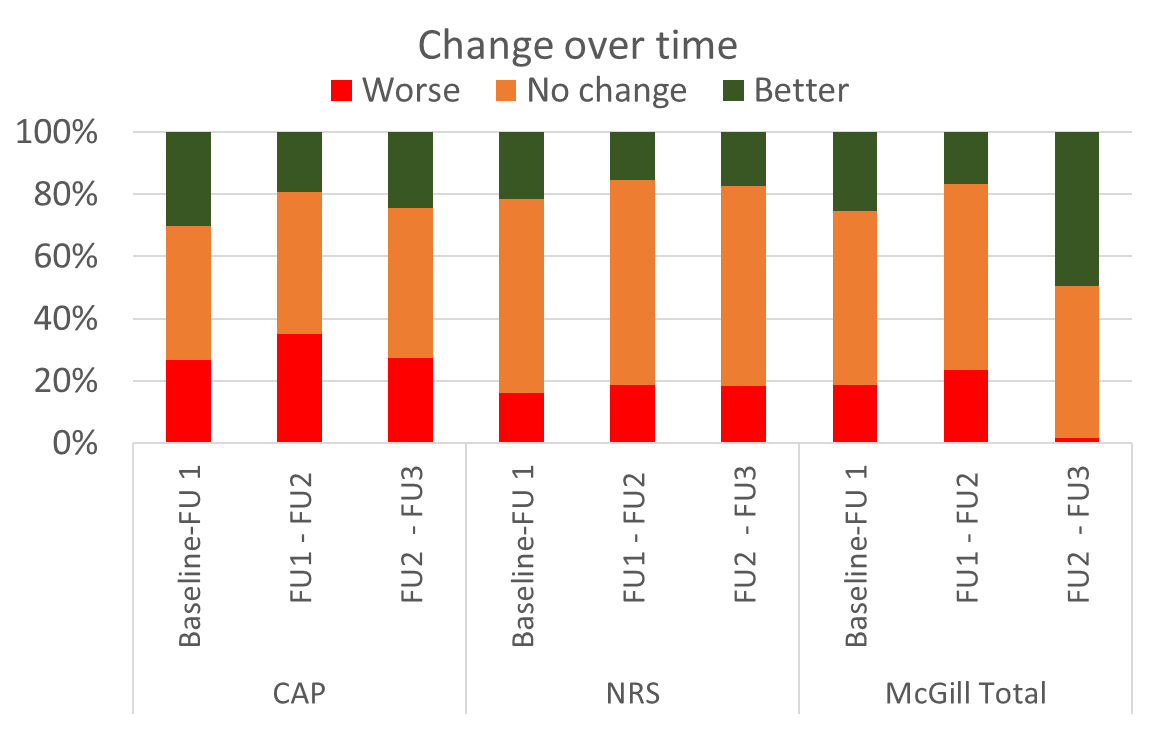


NRS – Numerical rating scale for Pain Intensity; FU1 – Follow-up year 1; FU2 – Follow-up year 2; FU3 – Follow-up year 3
